# Enduring Effects of Humanin on Mitochondrial Systems in TBI Pathology

**DOI:** 10.3390/biom15121705

**Published:** 2025-12-06

**Authors:** Pavan Thapak, Zhe Ying, Fernando Gomez-Pinilla

**Affiliations:** 1 Department of Integrative Biology and Physiology, University of California Los Angeles, Los Angeles, CA 90095, USA; 2 Department of Neurosurgery, University of California Los Angeles, Los Angeles, CA 90095, USA

**Keywords:** traumatic brain injury, cognition, mitochondrial dynamics, cellular energy, inflammation

## Abstract

Traumatic brain injury has long-term detrimental effects on neurological function and general quality of life of affected individuals. Bioenergetic failure is a primary mechanism for cellular dysfunction. We used the mitochondrial activator humanin (HN) to try to normalize the disruptive action of TBI on cellular bioenergetics in the hippocampus. We found that HN supplied right after the injury counteracted the action of TBI on metabolic sensing proteins (LKB1, AMPK, and AKT). HN also counteracted cognitive function and restored the synaptic proteins (Synapsin I and PSD-95) at three weeks post-injury. Moreover, HN normalized the disruptive action of TBI on mitochondrial functioning and dynamics (fusion, fission, and mitophagy). In addition, HN treatment counteracted TBI’s effects on mitochondrial biogenesis (PGC-1α), antioxidant (SOD2), and apoptotic marker (CC3). Furthermore, HN intervention in injured animals counteracted the gene expression linked with inflammation (*Itgax*, *SALL1*, *GFAP*, and *NLRP3*), synaptic plasticity (*HDAC2*), and bioenergetics (*mtND2*, *TFAM*, *SIRT1*, and *SIRT3*). These observations emphasize the therapeutic potential of HN by normalizing the fundamental aspects of TBI pathogenesis central to cellular bioenergetics and synaptic plasticity.

## 1. Introduction

Traumatic brain injury is a long-term debilitating condition that enhances metabolic demands of the brain and reduces the threshold for psychiatric disorders [1]. On a global scale, millions of people suffer brain injury every year [2,3]. TBI affects the metabolic ability of neuronal cells to sustain cellular functions. Effective therapeutic strategies are limited due to the complexity of TBI pathology. The reduction in cellular energy in TBI pathology unleashes a myriad of intermediate factors, resulting in synaptic dysfunction and cognitive decline [4]. Therefore, a desirable therapeutic approach is to normalize disrupted molecular events underlying bioenergetics.

The mitochondrial peptide humanin (HN) exhibits a range of actions centered on energy metabolism, which are achieved by binding to the CNTFR-α/gp-130/WSX-1 trimeric receptor [5,6,7,8,9,10]. HN is receiving additional attention because of its potential therapeutic action in the management of TBI [11,12,13] and other neurological disorders, such as Alzheimer’s disease [11,13]. We have recently reported that HN supplied immediately after the lesion ameliorates the short-term effects of TBI in mitochondrial activity up to one-week post-TBI [14]. This is significant since metabolic depression is a feature of brain injury pathology that reduces cell function, functional recovery, and cognitive function [15,16]. The regulation of energy metabolism at the cellular level is an evolutionarily conserved function of protein kinase B (AKT) and AMP-activated protein kinase (AMPK) by regulating catabolism mechanisms, including autophagy [17,18]. The metabolic stress state triggers the activation of liver kinase B1 (LKB1), which subsequently activates AMPK, a key regulator of cellular bioenergetics, autophagy, and cell survival [19]. Given the therapeutic potential of HN shown at the acute phase of TBI [14], it is important to determine the long-term effects of HN on TBI pathology. In addition, it is crucial to determine the impact of HN on molecular events secondary to the dysfunction of mitochondrial dynamics that can alter synaptic plasticity and cognitive function after TBI. Mitochondrial dysfunction is a central issue in energy deprivation that has subsequent effects on several cellular and molecular abnormalities, such as mitophagy. Mitophagy selectively eliminates mutilated mitochondria and helps to restore neuronal functioning [20,21]. Hence, the current study aims to examine the effects of HN on chronic TBI pathogenesis and investigate its potential to improve mitochondrial homeostasis and cognitive function.

## 2. Materials and Methods

### 2.1. Animals

**Figure 1:**
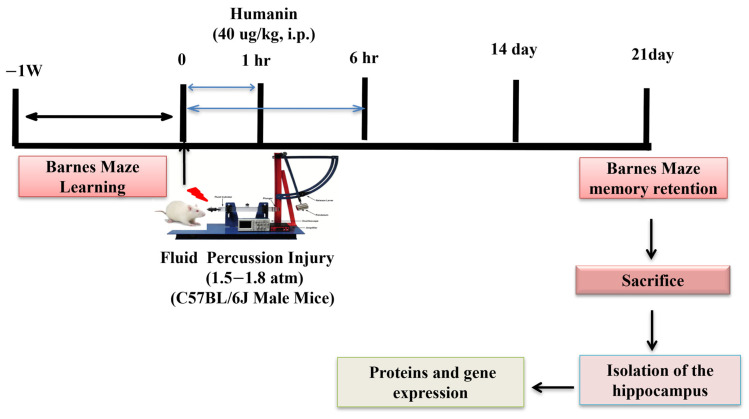
Experimentaldesign and HN treatment schedule. All animals completed two learning trials per day in the BM test for four consecutive days. Then, all animals were subjected to either sham or fluid percussion injury (FPI). HN (40 μg/kg, i.p) was administered at 1 and 6 h post-injury. Memory retention was assessed on the 21st day post-TBI. Then, the animals were sacrificed, and the ipsilateral hippocampus was isolated for biochemical and expression studies.

All experiments were approved by the Animal Research Committee, University of California, Los Angeles, in compliance with the United States National Institutes of Health Guide for the Care and Use of Laboratory Animals. C57BL/6J mice (10 weeks old) were housed under 12 h light and dark cycles with access to food and water ad libitum. Animals were kept in the vivarium for 7 days for acclimatization. All mice were randomly divided into groups before completing the learning trials. All mice were subjected to Barnes maze learning for 4 days. Then, mice received moderate fluid percussion injury or sham injury and were randomly divided into the following groups: sham animals treated with vehicle (Sh + Veh), TBI animals treated with vehicle (TBI + Veh), and TBI animals treated with humanin (TBI + HN). Humanin (HN, 40 μg/kg, i.p, MilliporeSigma, Burlington, MA, USA) was administered post-TBI at 1 and 6 h (Figure 1) [14,22]. The rationale for choosing the HN dose (40 μg/kg) was based on a previous in vivo study using HN. Moreover, TBI leads to mitochondrial dysfunction, resulting in reduced bioenergetic capacity at 1 h and 6 h, with a continuous decline up to 72 h post-TBI [22,23]. Vehicle-treated animals received saline. Memory retention was performed on the 21st day post-TBI. All mice were humanely harvested, and tissues were isolated for mechanistic studies.

### 2.2. Fluid Percussion Injury

Injury was induced by the previously described method [15]. In brief, the animals were anesthetized by 4% isoflurane, and 2% isoflurane was used to maintain an unconscious state during the surgery on a stereotaxic frame. An eye ointment was applied, and the animals were kept on a thermal pad to maintain their body temperature at 37 °C during TBI or sham procedures. A midline incision was made to expose the skull, and a 3 mm diameter craniotomy was performed on the left parietal cortex (from bregma: AP—2.5 mm; ML—2 mm) via a hand trephine. An injury hub made of plastic (4 mm) was placed around the craniotomy and sealed with dental cement. The injury hub was filled with sterile normal saline. Anesthesia was discontinued, and the injury hub was connected to the fluid reservoir. Mice received a moderate fluid-wave impact, equal to 1.5 ± 0.2 atm of pressure. An anesthesia system was reinstated to suture the skin. An antibiotic was applied to the wound site, and the animals were transferred to a home cage. Carprofen (pain killer) was injected subcutaneously in mice 45 min pre-TBI and 24 h post-TBI at a dose of 5 mg/kg for 3 days. The sham animals underwent the same surgical procedure except for the injury.

### 2.3. Barnes Maze Test

Memory function was assessed using the Barnes maze, which is a circular plate 120 cm in diameter containing 20 evenly spaced circular holes, 5 cm in diameter, around the edge. An escape box was placed underneath one hole, and the position of the box was constant. All animals completed two trials per day for four consecutive days. The time interval between the two trials was 15 min. All trials were conducted in the presence of uniformly illuminated bright lights, which act as aversive stimuli. All animals were placed at the center of the Barnes maze, and the test ended when the animals entered the box or 300 s had elapsed. After the learning trial, all animals received fluid percussion injury (FPI) or sham injury. After 21 days of FPI, memory retention was assessed, and latency to find the escape box was recorded by Any-maze software (version 5.1,Stoelting Co., Wood Dale, IL, USA). The apparatus surface was cleaned with 70% alcohol to eliminate olfactory signs, and four distinct cues (shapes) were used during the trials [15].

### 2.4. RT-PCR

Total RNA was isolated from the ipsilateral to the injury hippocampus using the E.Z.N.A^®^ Total RNA Kit (Omega Bio-Tek Inc., Norcross, GA, USA). According to the manufacturer’s instructions, cDNA (1 µg) was synthesized using the High-Capacity cDNA Synthesis Kit (Thermo Fisher Scientific, Waltham, MA, USA). Quantitative real-time PCR amplification was performed using a power SYBER green PCR master mix (Applied Biosystem, Waltham, MA, USA) and specific oligonucleotides to determine the relative gene expression on a CFX96 analyzer (Bio-Rad Laboratories Inc., Hercules, CA, USA). The thermal cycling profile featured a preincubation at 95 °C for 10 min, followed by 40 cycles of denaturation at 95 °C for 15 s, elongation at 65 °C for 60 s, and a melting curve (65 °C for 5 s and 95 °C 15 s) was subsequently generated. Actin was used for the normalization of genes. The 2^−ΔΔCT^ approach was used to determine the relative expression. Primers for the cDNA amplification are listed in Table 1 [24].

### 2.5. Western Blot Analysis

The ipsilateral hippocampus was homogenized in a RIPA lysis buffer (Thermo Scientific, Waltham, MA, USA) with a 1 μL/mL protease inhibitor cocktail. Protein concentration was determined by the BCA protein assay (Thermo Scientific, Waltham, MA, USA). Equal amounts of protein were separated with SDS-PAGE (10%) and transblotted to a PVDF membrane. After blocking with 5% BSA, the membrane was incubated with primary antibody, p-Akt (Cell Signaling Technology, Danvers, MA, USA, Cat#4058S), Akt (Cell Signaling Technology, Danvers, MA, USA, Cat#9272S), p-AMPK (Cell Signaling Technology, Danvers, MA, USA, Cat#2535S), AMPK (Cell Signaling Technology, Danvers, MA, USA, Cat#2532S), LKB1 (Cell Signaling Technology, Danvers, MA, USA, Cat#3047S), PSD95 (Cell Signaling Technology, Danvers, MA, USA), p-STAT3 (Tyr705) (Cell Signaling Technology, Danvers, MA, USA, Cat#4113S), STAT3 (Cell Signaling Technology, Danvers, MA, USA, Cat#9139S), OXPHOS cocktail (Thermo Fisher Scientific, Waltham, MA, USA, Cat#45-8099), MFN2 (Cell Signaling Technology, Danvers, MA, USA, Cat#9482S), DRP1 (Cell Signaling Technology, Danvers, MA, USA, Cat#8570S), OPA1 (Cell Signaling Technology, Denvers, MA, USA, Cat#80471S), LC3B (Cell Signaling Technology, Denvers, MA, USA, Cat#4108S), PINK1 (Santacruz Biotechnology, Dallas, TX, USA, Cat#sc-517,353), parkin (Santacruz Biotechnology, Dallas, TX, USA, Cat#sc-32,282), synapsin I (Santa Cruz Biotechnology, Dallas, TX, USA, Cat#sc-376,623), GFAP (Santacruz Biotechnology, Dallas, TX, USA, Cat#sc-33,673), SOD2 (Cell Signaling Technology, Denvers, MA, USA, Cat#13141S, 1:2000), PGC-1α (Invitrogen, Waltham, MA, USA, Cat#PA5–72948, 1:2000), CC3 (Cell Signaling Technology, Denvers, MA, USA, Cat#9664, 1:600), and actin (Santacruz Biotechnology, Dallas, TX, USA, Cat#sc-47778) overnight at 4 °C. After washing with TBST, the membrane was incubated with HRP-conjugated secondary antibody for 1 h at room temperature. After washing with TBST, the membrane was incubated with ECL to visualize bound antibodies using the gel doc. ImageJ (version 2.16) was used to quantify band intensity [25].

### 2.6. Statistical Analysis

The values were expressed as mean ± SEM. One-way analysis of variance (ANOVA), followed by Sidak’s post hoc multiple comparison test, was used to determine the significant differences among various groups. Graph Pad Prism 8 (version 9.3.0, San Diego, CA, USA) was used for statistical data analyses. *p* < 0.05 was considered statistically significant.

## 3. Results

### 3.1. Effect of HN on Cognitive Function Post-TBI

**Figure 2:**
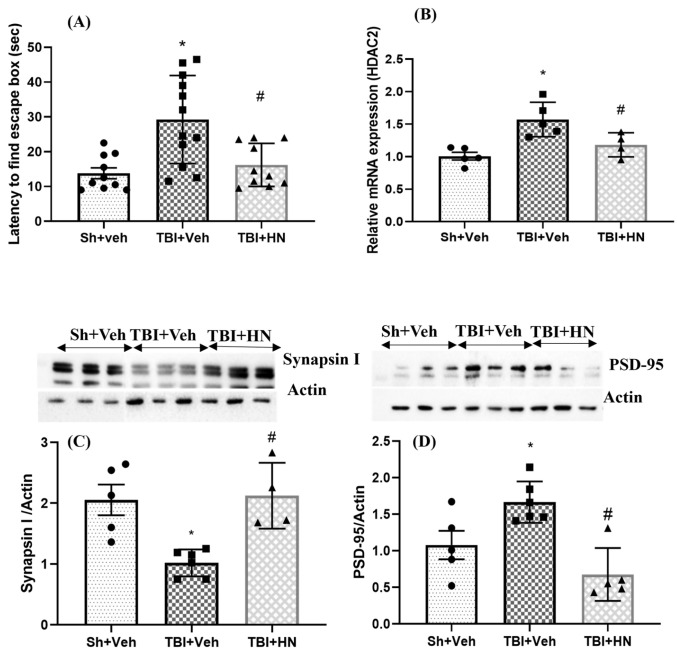
Effects of HN on cognitive assessment at three weeks post-TBI. (**A**) Latency to find the escape box in the BM on the 21st day post-TBI. (**B**) Relative mRNA expression of *HDAC2* in the hippocampus. (**C**) Protein levels of Synapsin I, (**D**) Protein levels of PSD-95 in the hippocampus. Values are expressed as mean + SEM, n = 4–12. Statistical significance was analyzed by one-way ANOVA, followed by Sidak’s post hoc multiple comparison test. * *p* < 0.05 vs. Sh + Veh animals, # *p* < 0.05 vs. TBI + Veh animals. WB original images can be found at Supplementary Materials.

The Barnes maze (BM) test was used to assess spatial memory in TBI-afflicted animals. All animals received learning trials for four consecutive days before TBI was performed. Three weeks after TBI, memory retention was performed and animals showed an increase in escape latency (time to find a hidden box; F_(2, 29)_ = 9.672, *p* = 0.0006; post hoc, *p* < 0.05) compared to sham animals (Figure 2A). However, HN intervention reduced (F_(2, 29)_ = 9.672, *p* = 0.0006; post hoc, *p* < 0.05) escape latency compared to TBI+Veh. In addition, we analyzed pre- and post-synaptic proteins that participate in synaptic plasticity in the hippocampus. Synapsin I levels decreased (F_(2, 12)_ = 10.28, *p* = 0.002; post hoc, *p* < 0.05) post-TBI, which was counteracted (F_(2, 12)_ = 10.28, *p* = 0.002; post hoc, *p* < 0.05) by HN intervention (Figure 2C). Synapsin I is a vesicle-associated protein that participates in vesicle docking at the synapse. PSD-95 is a postsynaptic protein and plays a crucial role in NMDA-mediated excitotoxicity. PSD-95 levels were increased (F_(2, 12)_ = 14.00, *p* = 0.0007; post hoc, *p* < 0.05) post-TBI, but then decreased (F_(2, 12)_ = 14.00, *p* = 0.0007; post hoc, *p* < 0.05) after HN administration (Figure 2D). Furthermore, we also analyzed HDAC2 gene expression, which modulates synapse formation. Hippocampal *HDAC2* mRNA levels were significantly increased (F_(2, 11)_ = 10.01, *p* = 0.003; post hoc, *p* < 0.05) post-TBI. HN treatment reduced *HDAC2* expression in the hippocampus post-TBI (Figure 2B).

### 3.2. Effects of HN on Metabolic Markers Post-TBI

**Figure 3:**
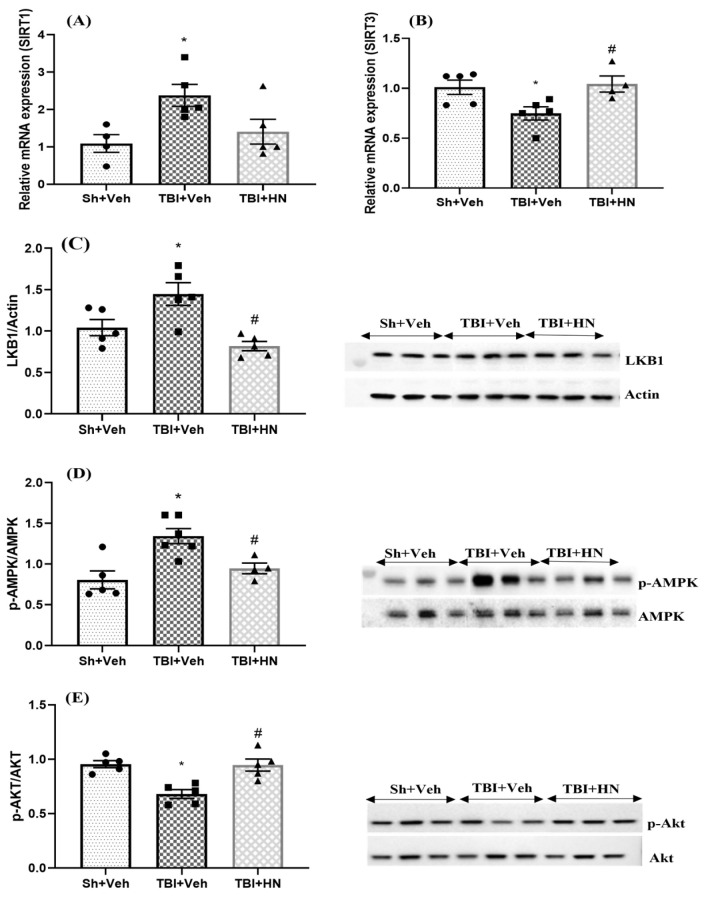
Effects of HN on cellular energy-sensing markers at three weeks post-TBI. Relative mRNA expression of (**A**) *SIRT1* and (**B**) *SIRT3*. Protein levels of (**C**) LKB1 (**D**) p-AMPK, and (**E**) p-AKT in the hippocampus. Values are expressed as mean + SEM, n = 4–6. Statistical significance was analyzed by one-way ANOVA, followed by Sidak’s post hoc multiple comparison test. * *p* < 0.05 vs. Sh + Veh animals, # *p* < 0.05 vs. TBI + Veh animals. WB original images can be found at Supplementary Materials.

We assessed the effects of HN on genes and proteins associated with cellular energy modulation. SIRT1 and SIRT3 are NAD^+^ histone deacetylases that sense energy deprivation and modulate metabolic processes. Gene expression of *SIRT1* increased (F_(2, 11)_ = 5.02, *p* = 0.02; post hoc, *p* < 0.05) while *SIRT3* was reduced (F_(2, 11)_ = 5.07, *p* = 0.02; post hoc, *p* < 0.05) post-TBI; however, HN treatment counteracted the post-TBI effects (Figure 3A,B). Additionally, AMPK and AKT are cellular proteins that sense metabolic activity. LKB1 is an upstream molecule that initiates AMPK phosphorylation in the glucose deprivation states. The TBI state increased LKB1 (F_(2, 12)_ = 9.643, *p* = 0.003; post hoc, *p* < 0.05) and p-AMPK (Thr-473) ((F_(2, 12)_ = 9.33, *p* = 0.003; post hoc, *p* < 0.05) while reducing p-AKT (Ser-473) (F_(2, 12)_ = 12.62, *p* =0.001; post hoc, *p* < 0.05) protein levels; however, treatment with HN significantly counteracted the effects of TBI (Figure 3C–E).

### 3.3. Effects of HN on Mitochondria Post-TBI

**Figure 4:**
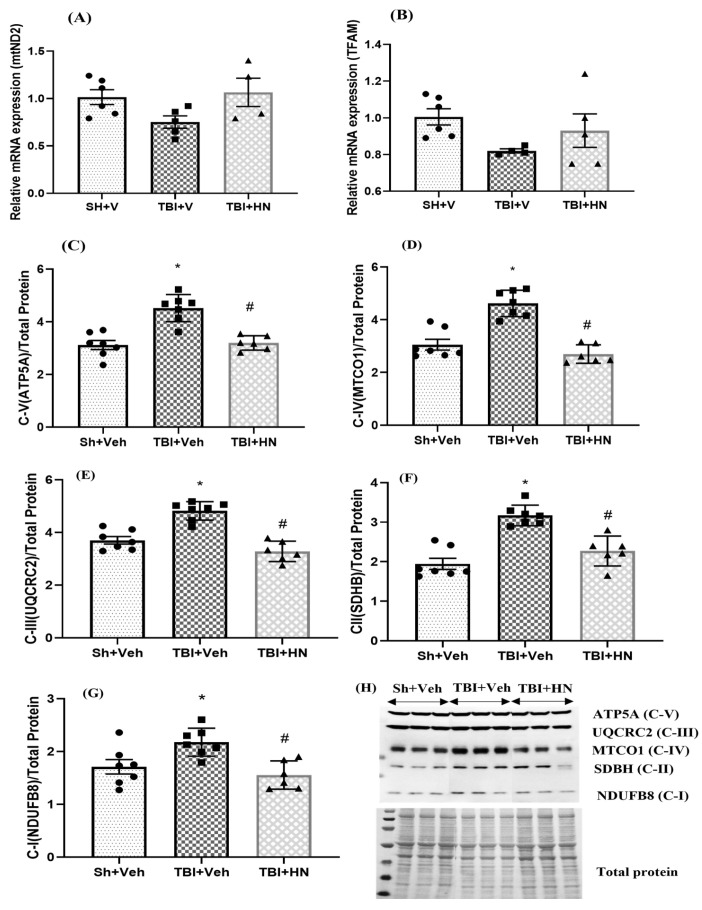
Effects of HN on mitochondrial OxPhos protein levels and gene expression at three weeks post-TBI. Relative mRNA expression of (**A**) *mtND2* and (**B**) *TFAM*. Immunoblot analysis of OXPHOS protein levels of (**C**) ATP5A (Complex-V), (**D**) MTCOI (Complex-IV), (**E**) UQCRC2 (Complex-III), (**F**) SDBH (Complex-II), and (**G**) NDUFB8 (Complex-I) in the hippocampus. (**H**) Representatives immunoblot of OXPHOS and total protein stained by Ponceau. Values are expressed as mean + SEM, n = 5–6. Statistical significance was analyzed by one-way ANOVA, followed by Sidak’s post hoc multiple comparison test. * *p* < 0.05 vs Sh + Veh animals, # *p* < 0.05 vs. TBI + Veh animals. [NDUFB8, NADH dehydrogenase beta subcomplex subunit 8 of Complex I; SDHB, succinate dehydrogenase subunit B of Complex II; MTCO1, cytochrome c oxidase subunit 1 of Complex IV; UQCRC2, cytochrome b-c1 complex subunit 2 of Complex III; ATP5A, ATP synthase subunit alpha of Complex V]. WB original images can be found at Supplementary Materials.

We assessed the effects of long-term TBI on genes and proteins associated with mitochondria. Mitochondrial gene *mt-ND2* (F_(2, 11)_ = 3.424, *p* = 0.06) and *TFAM* (F_(2, 12)_ = 2.183, *p* = 0.155) expression showed a trend to decreased expression post-TBI, which was normalized by HN intervention (Figure 4A,B). However, mitochondrial OxPhos Complex I (F_(2, 17)_ = 7.550, *p* = 0.004; post hoc, *p* < 0.05), Complex II (F_(2, 17)_ = 24.50, *p* = 0.0001; post hoc, *p* < 0.05), Complex III (F_(2, 17)_ = 30.61, *p* = 0.0001; post hoc, *p* < 0.05), Complex IV (F_(2, 17)_ = 30.69, *p* < 0.0001; post hoc, *p* < 0.05), and Complex V (F_(2, 17)_ = 22.41, *p* = 0.0001; post hoc, *p* < 0.05) protein levels were upregulated in the hippocampus post-TBI. HN intervention normalized the OXPHOS protein levels (*p* < 0.05) and counteracted the effects of TBI (Figure 4C–H).

### 3.4. Effects of HN on Markers Associated with Mitochondrial Fusion, Fission, and Mitophagy Post-TBI

**Figure 5:**
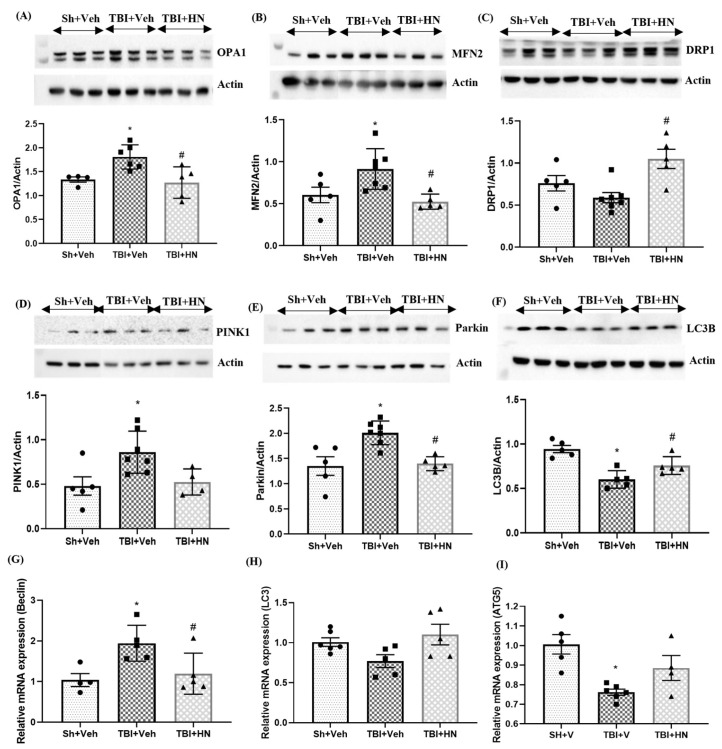
Effects of HN on mitochondrial fusion, fission, and mitophagy-associated protein levels at three weeks post-TBI. Immunoblot analysis of (**A**) OPA1, (**B**) MFN2, (**C**) DRP1, (**D**) PINK1, (**E**) parkin and (**F**) LC3B. Relative mRNA expression of (**G**) Beclin (**H**) LC3B, and (**I**) ATG5 in the hippocampus. Values are expressed as mean + SEM, n = 4–6. Statistical significance was analyzed by one-way ANOVA, followed by Sidak’s post hoc multiple comparison test. * *p* < 0.05 vs. Sh + Veh animals, # *p* < 0.05 vs. TBI + Veh animals. WB original images can be found at Supplementary Materials.

Mitochondrial fusion, fission, and mitophagy are crucial consequences of loss of mitochondrial homeostasis. The optic atrophy gene 1 (OPA1), mitofusion 2 (MFN2), and dynamin-related protein 1 (DRP1) proteins are essential components of mitochondrial fusion and fission. OPA1 (F_(2, 12)_ = 7.12, *p* = 0.009; post hoc, *p* < 0.05) and MFN2 (F_(2, 12)_ = 10.41, *p* = 0.002; post hoc, *p* < 0.05) protein levels were significantly increased (*p* < 0.05) post-TBI (Figure 5A,B). However, HN intervention counteracted the TBI effects by normalizing mitochondrial fusion protein dynamics. The HN intervention reduced the mitochondrial fission protein DRP1 level (F_(2, 14)_ = 7.387, *p* = 0.006), which was increased by HN intervention (Figure 5C).

PINK1 and parkin are essential proteins that participate in mitophagy. PINK1 (F_(2, 12)_ = 6.48, *p* = 0.013; post hoc, *p* < 0.05) and parkin (F_(2, 14)_ = 10.72, *p* = 0.001; post hoc, *p* < 0.05) protein levels were increased, while LC3β (F_(2, 12)_ = 15.69, *p* = 0.0004; post hoc, *p* < 0.05), an autophagosome marker, was reduced post-TBI (Figure 5D–F). Moreover, *Beclin* (F_(2, 11)_ = 5.781, *p* = 0.019; post hoc, *p* < 0.05) gene expression was increased, while *LC3B* (F_(2, 13)_ = 2.23, *p* = 0.14) and *ATG5* (F_(2, 12)_ = 9.249, *p* = 0.003; post hoc, *p* < 0.05) gene expression was reduced post-TBI (Figure 5G–I). However, HN intervention counteracted the effects of TBI on mitochondrial fusion, fission, and mitophagy.

### 3.5. Effects of HN on Microglia and Astrocytes Post-TBI

**Figure 6:**
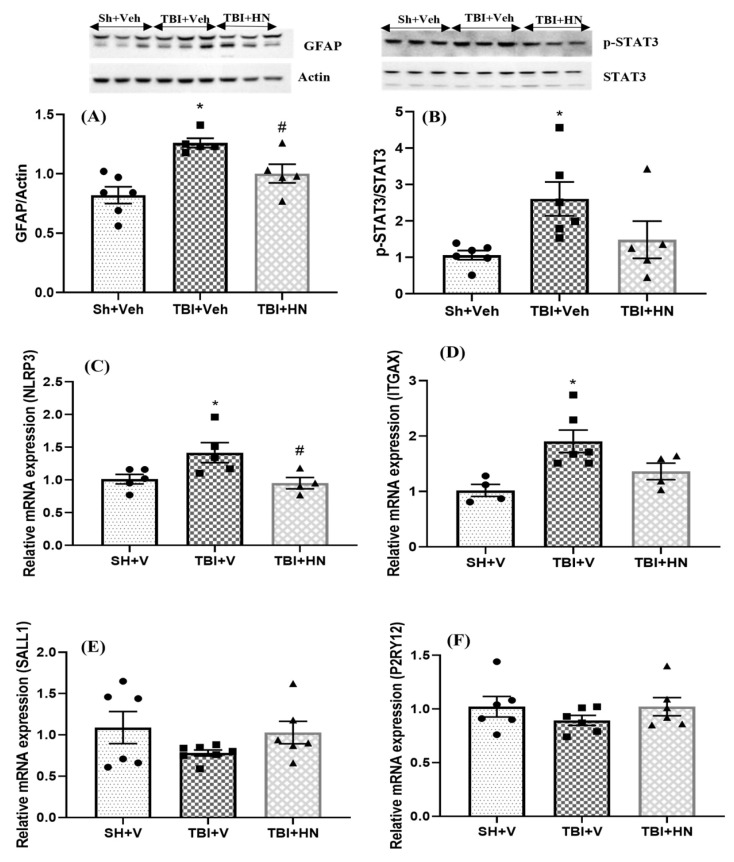
Effects of HN on microglial and astrocyte homeostasis. Immunoblot analysis of (**A**) GFAP and (**B**) p-STAT3. Relative mRNA expression of (**C**) *NLRP3*, (**D**) *ITGAX*, (**E**) *SALL1*, and (**F**) *P2RY12* in the hippocampus. Values are expressed as mean + SEM, n = 4–6. Statistical significance was analyzed by one-way ANOVA, followed by Sidak’s post hoc multiple comparison test. * *p* < 0.05 vs. Sh + Veh animals, # *p* < 0.05 vs. TBI + Veh animals. WB original images can be found at Supplementary Materials.

Microglia and astrocytes have essential roles in the maintenance of brain homeostasis and plasticity. GFAP (F_(2, 13)_ = 11.42, *p* = 0.001; post hoc, *p* < 0.05) and p-STAT3 (F_(2, 14)_ = 4.371, *p* = 0.031; post hoc, *p* < 0.05) (Figure 6A,B) protein levels were increased post-TBI, while HN intervention counteracted these increases. Microglial homeostatic genes (*SALL1* (F_(2, 14)_ = 2.895, *p* = 0.088) and *P2ry12* (F_(2, 14)_ = 1.88, *p* = 0.188)) (Figure 6E,F) tended to decrease post-TBI. Moreover, *NLRP3* (F_(2, 11)_ = 5.022, *p* = 0.028; post hoc, *p* < 0.05) and *ITGAX* (F_(2, 11)_ = 9.971, *p* = 0.003; post hoc, *p* < 0.05) (Figure 6C,D) gene expression was upregulated post-TBI, which is associated with activated microglia and inflammation, respectively.

### 3.6. Effects of HN on Mitochondrial Biogenesis, Antioxidant, and Apoptotic Marker Post-TBI

**Figure 7:**
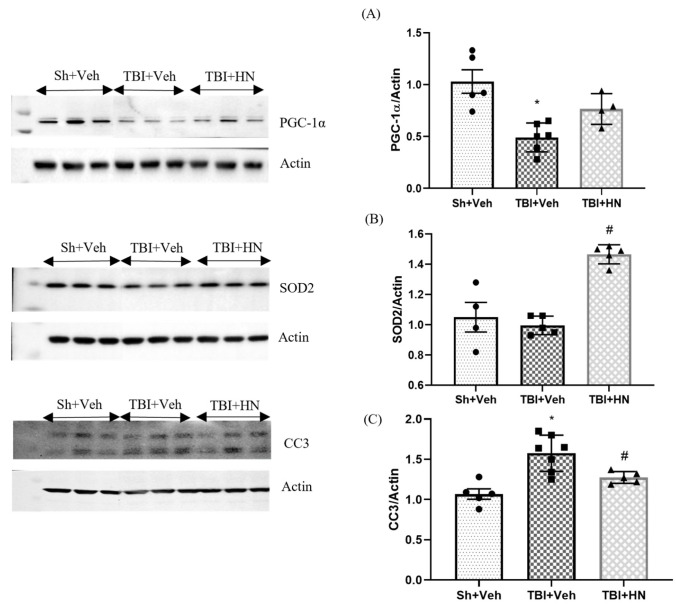
Effects of HN on mitochondrial biogenesis, antioxidant, and apoptotic markers at 3 weeks post-TBI. Immunoblot analysis of (**A**) PGC-1α, (**B**) SOD2, (**C**) CC3 in the hippocampus. Values are expressed as mean + SEM, n = 4–6. Statistical significance was analyzed by one-way ANOVA, followed by Sidak’s post hoc multiple comparison test. * *p* < 0.05 vs. Sh + Veh animals, # *p* < 0.05 vs. TBI + Veh animals. WB original images can be found at Supplementary Materials.

We further measured the protein level of PGC-1α, a transcriptional factor involved in mitochondrial biogenesis. TBI vehicle reduced levels of PGC-1α (F_(2, 12)_ = 11.36, *p* = 0.0007; post hoc, *p* < 0.05) as compared to sham animals, while HN administration showed a trend to increased PGC-1α levels (F_(2, 12)_ = 9.069, *p* = 0.002) in TBI-afflicted animals (Figure 7A). Furthermore, we assessed the mitochondrial antioxidant marker SOD2, which neutralizes the reactive oxygen species generated in mitochondria during ATP synthesis. The protein level of SOD (F_(2, 11)_ = 24.26, *p* = 0.0001) was unchanged post-TBI at 3 weeks as compared to sham animals. However, HN administration enhanced the SOD level (F_(2, 11)_ = 24.26, *p* = 0.0001) in TBI animals (Figure 7B). Moreover, we observed a significant increase in the cleaved caspase 3 (CC3) (F_(2, 14)_ = 13.43, *p* = 0.0006; post hoc, *p* < 0.05) (Figure 7C) protein level post-TBI. CC3 is a central effector for apoptosis, which is directly linked to neuronal injury [26] and promotes the inflammatory process [27]. However, HN intervention neutralized the TBI effects.

## 4. Discussion

**Figure 8:**
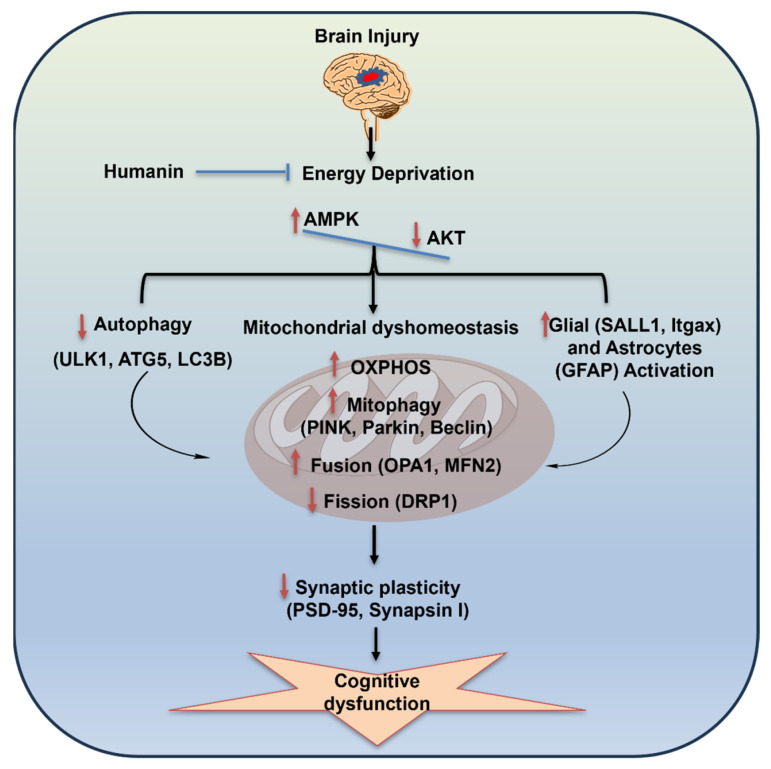
Schematic overview of the promising neuroprotective effects of HN on long-term TBI pathology. The TBI state modulates cellular energy sensing proteins (AMPK and AKT) and disrupts mitochondrial homeostasis. Furthermore, a long-term TBI state interrupts the mitochondrial quality system by upregulating fusion and mitophagy markers while reducing fission and autophagy markers. Moreover, the TBI state triggers the activation of microglial and astrocyte cells associated with the inflammatory response. Alterations in the neuronal metabolic state and inflammatory cells modulate synaptic integrity by modulating synaptic proteins (synapsin I and PSD-95), resulting in cognitive decline. However, HN treatment restores cognitive function by neutralizing the long-term post-TBI effects. Overall, this study illustrates the speculated mechanism through which HN intervention restores mitochondrial homeostasis and cognitive function by neutralizing the impact of long-term post-TBI.

TBI pathology is accompanied by brain metabolic abnormalities that compromise cell survival and function, making it crucial to develop a therapeutic strategy that supports cellular bioenergetics. The mitochondrial peptide humanin has recently emerged as a promising tool to enhance bioenergetics during the acute phase of TBI [14,28]. Here, we show for the first time that HN administration within the first 6 h post-injury exerts long-lasting effects that persist for three weeks after TBI. We provide new evidence on the action of HN on mitochondrial quality control processes, which are deeply involved in restoring bioenergetics and supporting cognitive function and mitochondrial homeostasis. Our findings support the therapeutic potential of HN in attenuating long-term consequences of TBI through acute-phase intervention (Figure 8).

### 4.1. Effects of HN on Metabolic Homeostasis After TBI

Mitochondrial function coordinates the balance between catabolism and anabolism to sustain the energy demands of cells [29]. We observed an increase in mitochondrial OxPhos complex proteins (C-I, C-II, C-III, C-IV, and C-V) levels three weeks after TBI, which were normalized by HN intervention. The precise molecular mechanism for upregulating OxPhos complex protein levels is still unclear. Alternatively, it may reflect an accumulation of non-functional mitochondrial complexes due to impaired proteolysis. Growing evidence suggests that TBI leads to impaired proteolysis [30,31]. Additionally, we observed the downregulation of the autophagy marker, which also triggered the accumulation of impaired mitochondria or non-functional mitochondrial complexes. Furthermore, we also observed a reduction in the biogenesis marker PGC1α (Figure 7A) following TBI. These observations further support the notion that these may have been mutilated mitochondria that accumulated due to the failure of cellular homeostasis following TBI. However, assessing functional mitochondrial activity is required to confirm that the increased mitochondrial complex proteins are indeed non-functional mitochondria following long-term TBI.

Furthermore, we measured the protein expression of SOD2, a mitochondrial antioxidant marker, and cleaved caspase 3 (CC3), an apoptotic marker. We observed that the SOD 2 levels after 3 weeks post-injury were unchanged compared to sham animals. A previous study showed that SOD2 levels were reduced up to 7 days after injury, while by the 14th day, this reversed [32]. Our findings are consistent with the previous observations. Moreover, the apoptotic marker cleaved caspase 3 (CC3) (Figure 7C) showed increased protein levels at 3 weeks post-injury in the hippocampus. Previous findings have also demonstrated that activation of caspase 3 lasts for 3 months following experimental CCI. A significant increase in cleaved-caspase-3-positive cells was observed at 1 month after injury [33,34]. In addition, clinical data indicate that chronic inflammation and white matter degeneration resulting from TBI can persist for years after even a single impact [35]. These observations suggest that CC3-mediated neuronal damage is accompanied by chronic inflammation, presumably involving the reactive astrocytes and microglia. However, HN intervention strongly reduces CC3 levels while increasing SOD2 levels in the hippocampus at 3 weeks post-injury. Taken together, these observations suggest that acute administration of HN has the potential to mitigate secondary pathogenesis after injury.

The brain is the most energy-consuming organ of the body, accounting for 20% of total energy expenditure, and mitochondrial bioenergetics is crucial for brain function [14,15]. In this study, mtND2 and TFAM mRNA levels were reduced post-TBI in agreement with their bioenergetic actions [36]. Chronic TBI leads to DNA hypermethylation at the *TFAM* promoter region, resulting in *TFAM* gene repression in the hippocampus. Reductions in TFAM levels associated with a decline in binding to mtDNA are tightly correlated with low energy (ATP) levels [36,37].

Metabolic dysfunction that frequently occurs following TBI appears to also involve the AMPK/AKT system. AMPK and AKT are responsive to metabolic stress and facilitate metabolic adjustments. AMPK is also known as an energy sensor that activates in response to ATP depletion to promote catabolism such as glucose uptake, fatty acid oxidation, and autophagy. AKT functions as an anti-apoptotic factor in response to numerous stimuli, such as hypoxia, which facilitates glycolysis and GLUT1 trafficking and elevates mitochondrial oxygen consumption [38,39]. Interestingly, both AMPK and AKT have crucial roles in metabolic homeostasis. Emerging evidence shows that glucose deprivation or metabolic stress leads to conformational changes in v-ATPase, allowing the docking of LKB1 and subsequently triggering AMPK phosphorylation [40,41,42,43]. Our findings indicate that TBI altered AKT, AMPK, and LKB1 protein levels; however, HN intervention mitigated TBI effects. These observations suggest that HN intervention promotes metabolic homeostasis, which is disrupted after TBI.

### 4.2. Effects of HN on Mitochondrial Dynamics; Bioenergetic, Fusion, Fission, and Mitophagy

It has been shown that mitochondria undergo persistent fusion and fission to meet cellular energy demand and preserve mitochondrial dynamics, as these can be detrimental to neuronal cell integrity [16,44,45]. Mitochondrial dynamics are regulated by a highly complex system known as mitochondrial quality control (MQC). Many conserved GTPase proteins, particularly optic atrophy 1(OPA1) and mitofusin (MFN2), are required for mitochondrial inner and outer membrane fusion, respectively, while dynamin-related protein 1 (DRP1) is required for mitochondrial fission dynamics [46]. During periods of high metabolic stress, mitochondria undergo hyperfusion, increasing cristae density to amplify energy production [47,48]. Our findings show that the OPA1 and MFN2 protein levels were significantly increased, while DRP1 protein levels were reduced post-TBI. Our observations indicate that a long-term TBI state promotes mitochondrial fusion to compensate for energy deprivation. However, HN intervention counteracted TBI effects by establishing mitochondrial dynamics.

Mitophagy is crucial to preserve cellular functions by eliminating damaged mitochondria post-TBI [46,49,50]. Alterations in mitochondrial fusion and fission led to mitophagy activation, evident by the increased mitophagy markers (PINK1, parkin, and BNIP3) post-TBI. These markers were neutralized by HN intervention in the current study. Earlier observations have shown that PINK1 accumulates on damaged mitochondria, further enhancing parkin recruitment [51,52]. However, autophagic markers (LC3β and ATG5) were reduced post-TBI, which was counteracted by HN treatment. These observations imply that disruption of the autophagy process may lead to the accumulation of mutilated mitochondria in cells, resulting in bioenergetic dysfunction. The classical theory suggests that mitochondrial fusion and mitophagy are coordinated and reciprocal processes that exist in homeostatic conditions [53]. However, a pathological state triggers the disturbance of this homeostatic cellular coordination. This may be due to the first attempt to rescue cells from energy deprivation, which might be the presence of heterogeneous mitochondria or non-specific activation of quality control systems. However, earlier findings showed that acute administration of HN neutralized energy deprivation [14] and promotes\d cellular homeostasis. Further exploratory studies are required to confirm the mitochondrial structure and colocalization of autophagic markers. Overall, these observations indicate that a long-term TBI state triggers dysregulation of mitochondrial homeostasis, while HN intervention applied the same day of the injury normalizes several dysregulations in the mitochondrial machinery post-TBI. Supported by our early genomic studies [22], it is likely that HN changes the program of genes after TBI. In addition, earlier observations suggested that HN treatment itself could induce intracellular HN expression and enhance mitochondrial biogenesis, inducing mitochondrial gene expression [54]. Moreover, in the current study, we also observed that HN intervention tended to increase mitochondrial genes, biogenesis markers, and cellular survival proteins [7]. Taken together, we can assertively suggest that HN has a long-term neuroprotective effect on brains affected by injury.

### 4.3. Effects of TBI and HN on Cognitive Function

We observed cognitive decline, evident from increased latency to find the escape box in the Barnes maze (BM) test three weeks after TBI. These extend previous results of a similar cognitive decline one week after TBI [14]. Furthermore, we also found a reduction in the pre-synaptic protein synapsin I and a reduction in the post-synaptic protein PSD-95, the effects of were counteracted by HN intervention. Synapsin I, a pre-synaptic protein regulates the anchoring of the vesicle linked with the release of neurotransmitters at the synaptic cleft and crucially participates in synapse formation. In turn, PSD-95, a guanylate kinase binds to the NMDA subunit GluN2B, leading to overstimulation of the NMDA receptor and an enhancement in neuronal vulnerability [55,56]. We also found that HDAC2 gene expression was increased post-TBI, which was counteracted by HN treatment. Earlier findings imply that over-expression of HDAC2 reduces dendritic spine density, resulting in reduced synapse formation, which triggers memory impairment [57]. These findings suggest that HN intervention improved cognitive deficits associated with long-term post-TBI.

### 4.4. Effects of HN on Microglial and Astrocytes Post-TBI

Microglia and astrocytes have various actions supporting neuronal function, including the regulation of synaptic pruning, nutrition, immune response, etc. Abundant evidence indicates that brain injury can alter the morphology and activity of astrocytes and microglia, contributing to restoring brain homeostasis [58,59]. Neuroinflammation is central to the pathological mechanisms of brain injury. Microglia cells are immune cells that regulate inflammation by drastically altering their phenotype and functions (M2 to M1 polarization). Earlier findings showed that the inflammatory response triggers the JAK2/STAT3 pathway in the pre-ischemic area. Microglia express many transcription regulators (JAK2/STAT3) that may lead to phenotypic transformation (M2 to M1) by affecting regulatory genes of microglia [60,61]. Moreover, suppressing homeostatic genes (*P2ry12*, *SALL1*) and the upregulation of *Itgax* (encoding the CD11c surface protein) have been associated with activated microglia, which executes an inflammatory and phagocytic response in a disease state [62,63]. In this study, we observed the downregulation of microglial homeostatic gene *SALL1*, while *Itgax* (Cd11c) surface protein gene expression was increased three weeks after TBI. Moreover, phosphorylation of STAT3 was increased post-TBI. These observations imply that the TBI state leads to phenotypic polarization of microglial cells (M2 to M1) by activating the STAT3 transcription factor.

Astrocytes also play a crucial role in the pathogenesis of brain injury. Astrocytes critically regulate synapse formation, the blood–brain barrier, fluid and ion homeostasis, and inflammation. Astrocytes respond to CNS injuries, including brain injury, and become activated, which induces molecular and morphological changes [59,64]. Our current study, as well as a previous study, showed that GFAP protein levels were increased in the hippocampus post-TBI, which was counteracted by HN intervention [14]. Increased GFAP protein levels are correlated with activated astrocytes (astrogliosis), which are also observed in TBI patients [65,66]. Additionally, astrogliosis potentiates immune responses, which promotes an infiltration of peripheral immune cells. Previous observations have shown that HN treatment counteracted pro-inflammatory cytokines, resulting in the inhibition of astrocytes and glial cell polarization, subsequently impeding neuronal damage [14,67]. Overall, HN intervention counteracted the harmful response of microglia and astrocytes and prevented neuronal damage post-TBI.

### 4.5. Study Limitations and Future Directions

In this study, functional or activity assays were not performed for the mitochondrial activity, oxidative stress activity, or caspase activity, which would provide more rigorous clarification of the chronic pathology of injury. In addition, immunohistological examination of mitochondrial structure, as well as colocalization of mitochondria with autophagy markers, would further provide the pathological involvement of mitochondrial dynamics post-injury. In addition, in this study, we did not observe the HN effects on different models of brain injury, such as closed head injury, which would further extend the therapeutic potential of humanin for the management of brain injury. Future studies are warranted to comprehensively assess pathological changes at 14 days after injury.

## 5. Conclusions

HN intervention effectively counteracts cognitive decline post-TBI, underscoring a long-term frame for both acute and chronic scenarios. Moreover, TBI pathology includes multifaceted cellular events, like synaptic plasticity, mitochondrial dysfunction, and the activation of microglia, astrocytes, and apoptotic markers, which are reduced by HN administration. The current findings show that HN intervention improves mitochondrial homeostasis, mitigating inflammation and enhancing cognitive capabilities, demonstrating the positive impact of HN on TBI pathogenesis. Post-TBI bioenergetic deprivation appears to be a significant catalyst for initiating other cellular events that contribute to secondary pathogenesis, which HN intervention effectively neutralizes based on both current and past reports. Overall, the effects of HN on cellular events, like synaptic plasticity and mitochondrial homeostasis, are crucial for the clinical management of long-term brain injury. HN intervention holds therapeutic promise for TBI treatment, as it restores essential cellular functions, particularly bioenergetic homeostasis.

## Figures and Tables

**Table 1:** List of primers used.

Primers	Forward	Reverse
*mt-ND2*	GGGTGTGAGGGATTTCATCGT	TGCATGGCTCTGGTTACCTC
*TFAM*	GTCGCATCCCCTCGTCTATC	TTTCTGGTAGCTCCCTCCACA
*HDAC2*	GTCTCGCTGGTGTTTTGCG	GCAGCCCTTCCATTTGAACC
*SIRT1*	CGGCTACCGAGGTCCATATAC	AAC ATG GCT TGA GGG TCT GG
*SIRT3*	GACTGGTCACGTAGCCTCAAG	ACAGAGGGATATGGGCCTTCT
*NLRP3*	CAGCGATCAACAGGCGAGAC	AGAGATATCCCAGCAAACCTATCCA
*Beclin*	GCCTCTGAAACTGGACACGA	TAGCCTCTTCCTCCTGGGTC
*SALL1*	TGTCAAGTTCCCAGAAATGTTCCA	ATGCCGCCGTTCTGAATGA
*ITGAX(Cd11c)*	CTGGATAGCCTTTCTTCTGCTG	GCACACTGTGTCCGAACTCA
*Actin*	ATGCTCCCCGGGCTGTAT	CATAGGAGTCCTTCTGACCCATTC

## Data Availability

The original findings in this study are included within the article. The data will be provided on a reasonable request to the corresponding author.

## Supplementary Materials

The following supporting information can be downloaded at https://www.mdpi.com/article/10.3390/biom15121705/s1: WB original images.
