# Supplementary material for: Enduring Effects of Humanin on Mitochondrial Systems in TBI Pathology
Source: Biomolecules. 2025 Dec 6;15(12):1705. doi: 10.3390/biom15121705 (PMC12730897; doi:10.3390/biom15121705)

Enduring Effects of Humanin on Mitochondrial Systems in TBI Pathology

Pavan Thapak [1], Zhe Ying [1] and Fernando Gomez-Pinilla [1,2,*]

1 Department of Integrative Biology and Physiology, University of California Los Angeles, Los Angeles, CA 90095, USA

2 Department of Neurosurgery, University of California Los Angeles, Los Angeles, CA 90095, USA

* Correspondence: fgomezpi@ucla.edu

Raw figure of Blots:

Fig.2 (C) Synapsin I

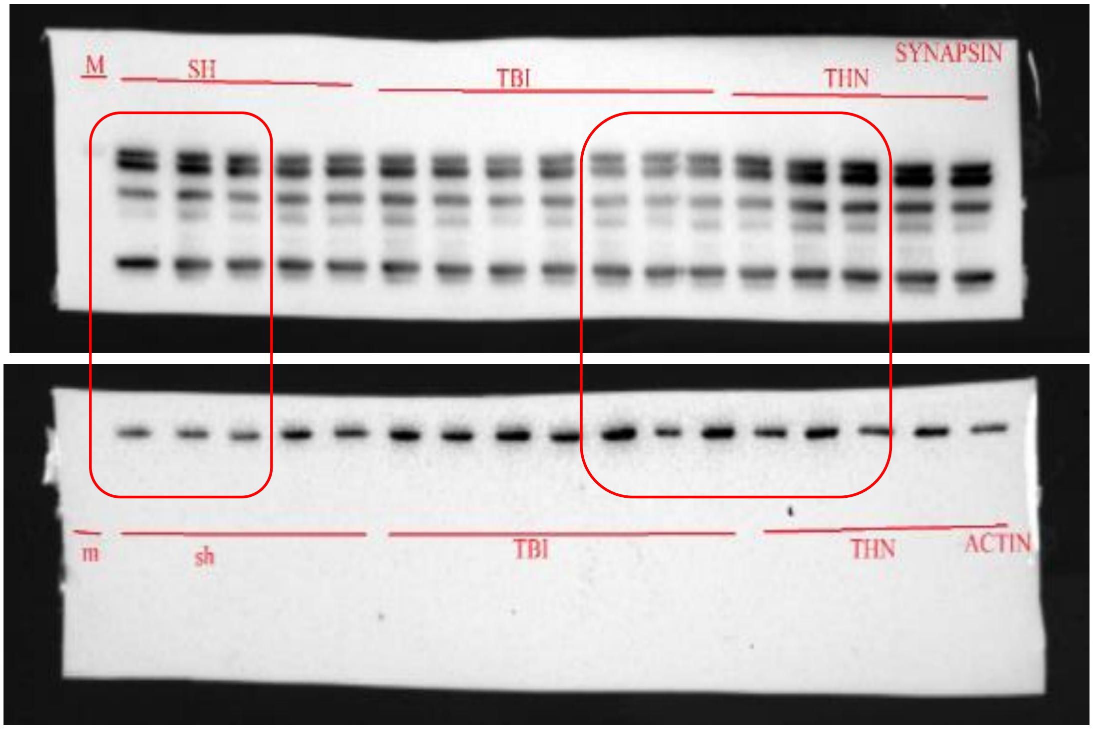

Fig.2 (D) PSD-95

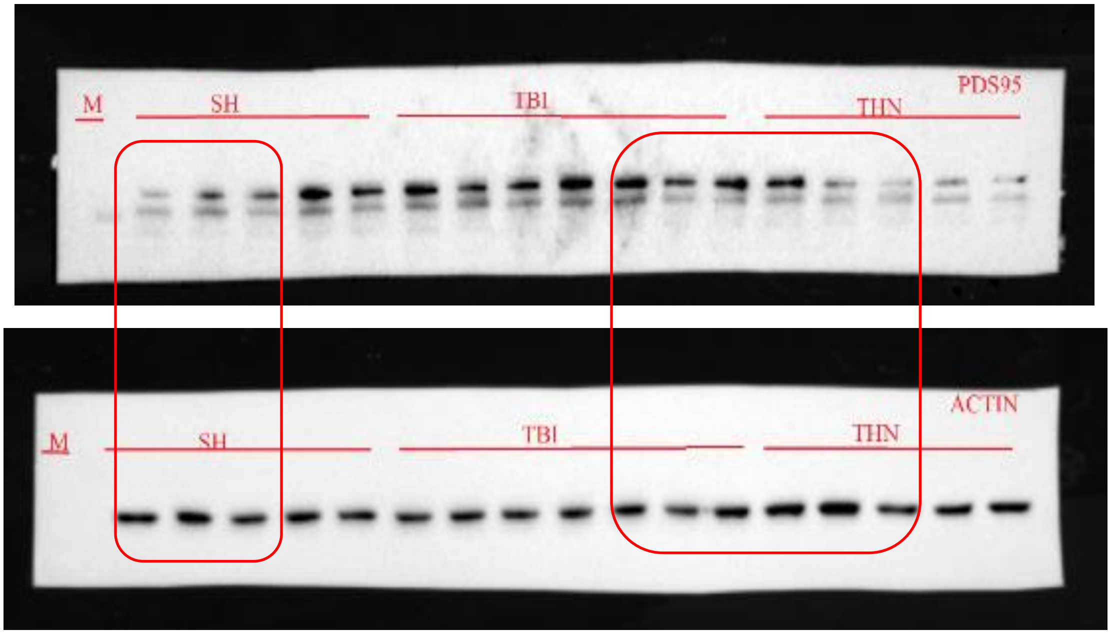

Fig.3 (C) LKB1

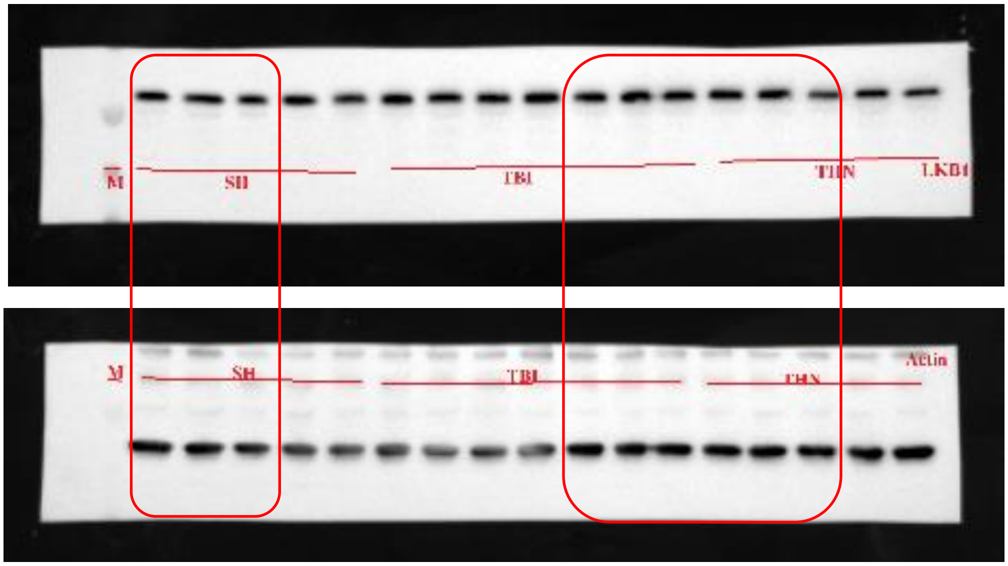

Fig.3 (D) p-AMPK

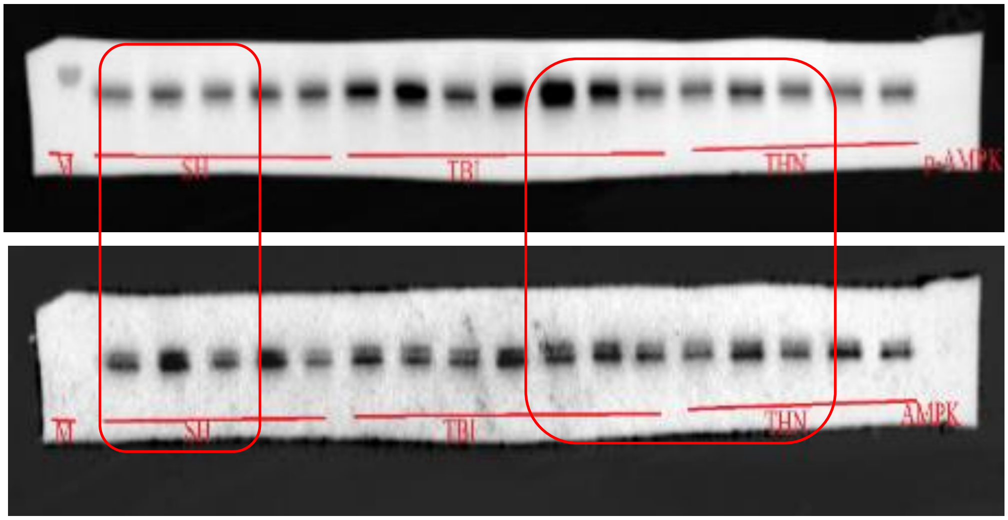

Fig.3 (E) p-AKT

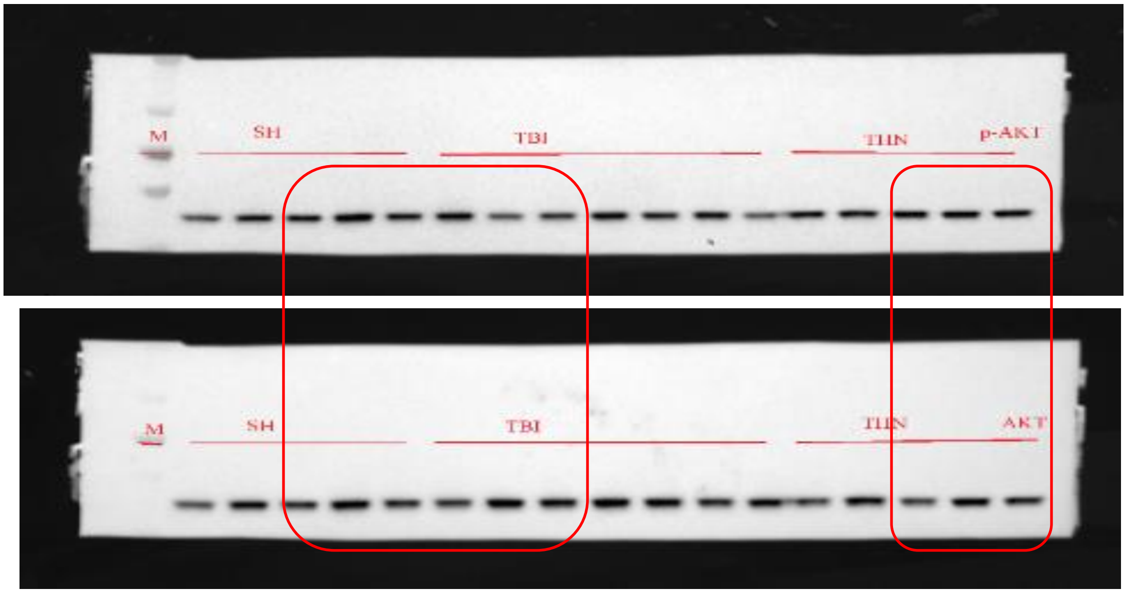

Fig.4 (H) OxPhos

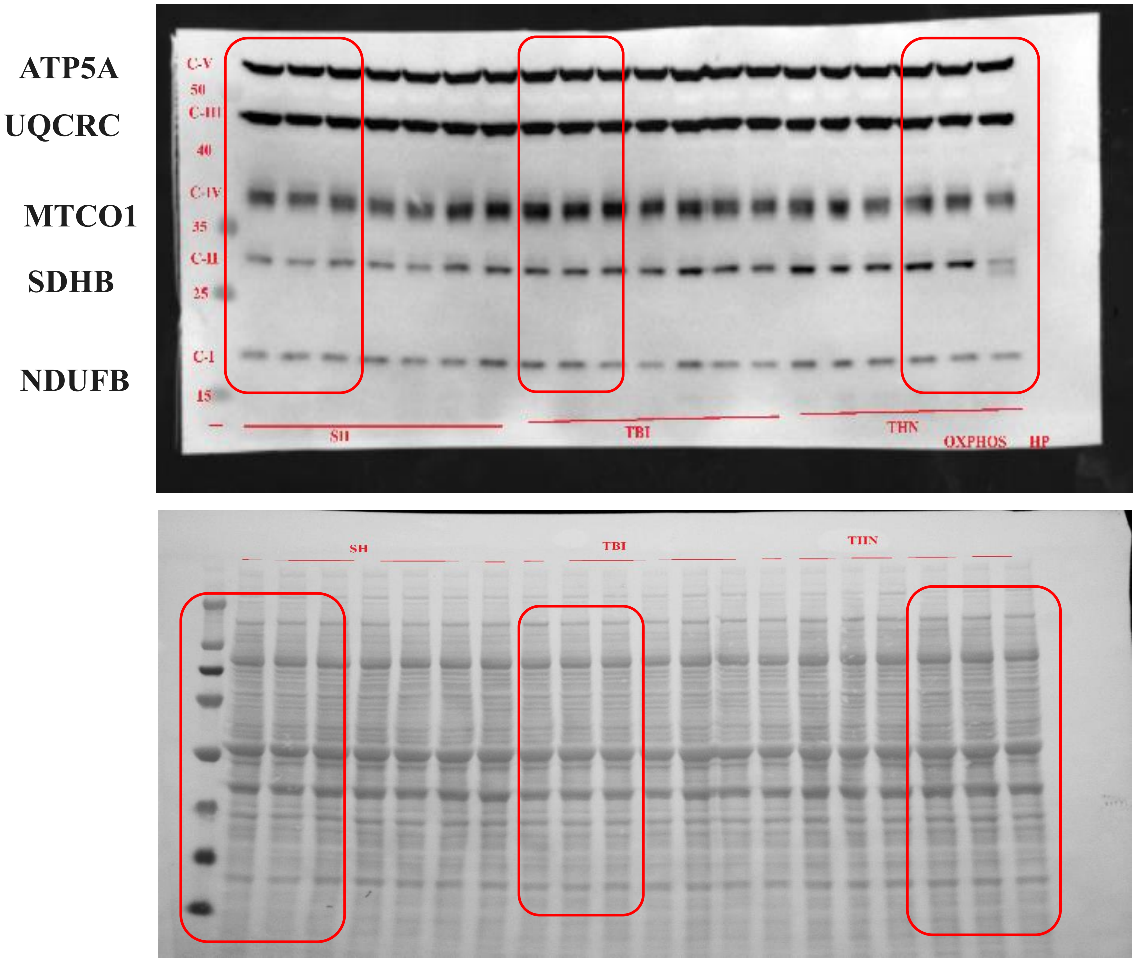

Fig.5 (A) OPA1

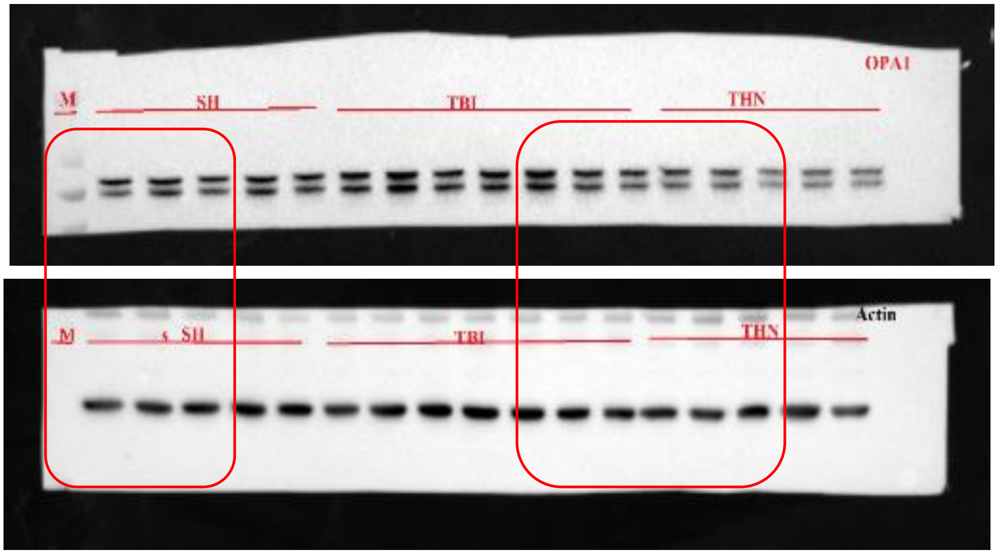

Fig.5 (B) MFN2

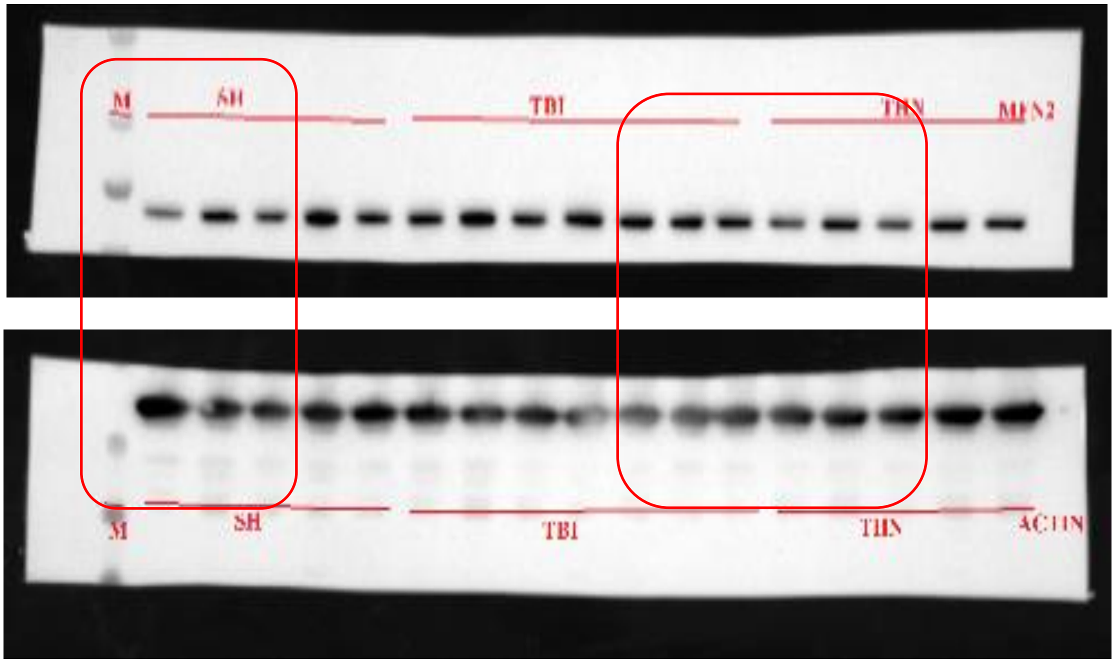

Fig.5 (C) DRP1

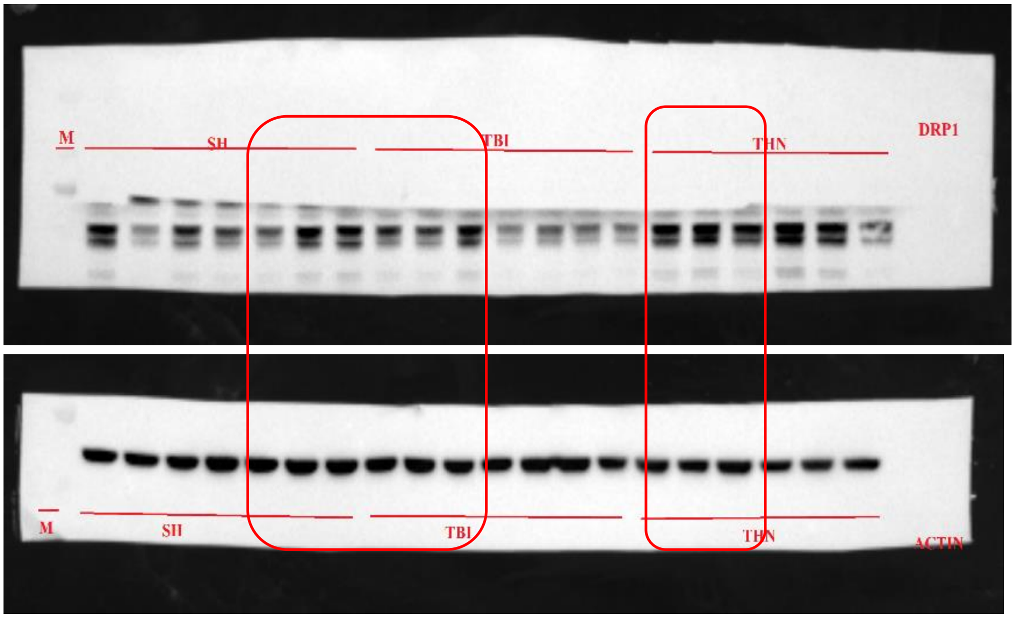

Fig.5 (D) PINK1

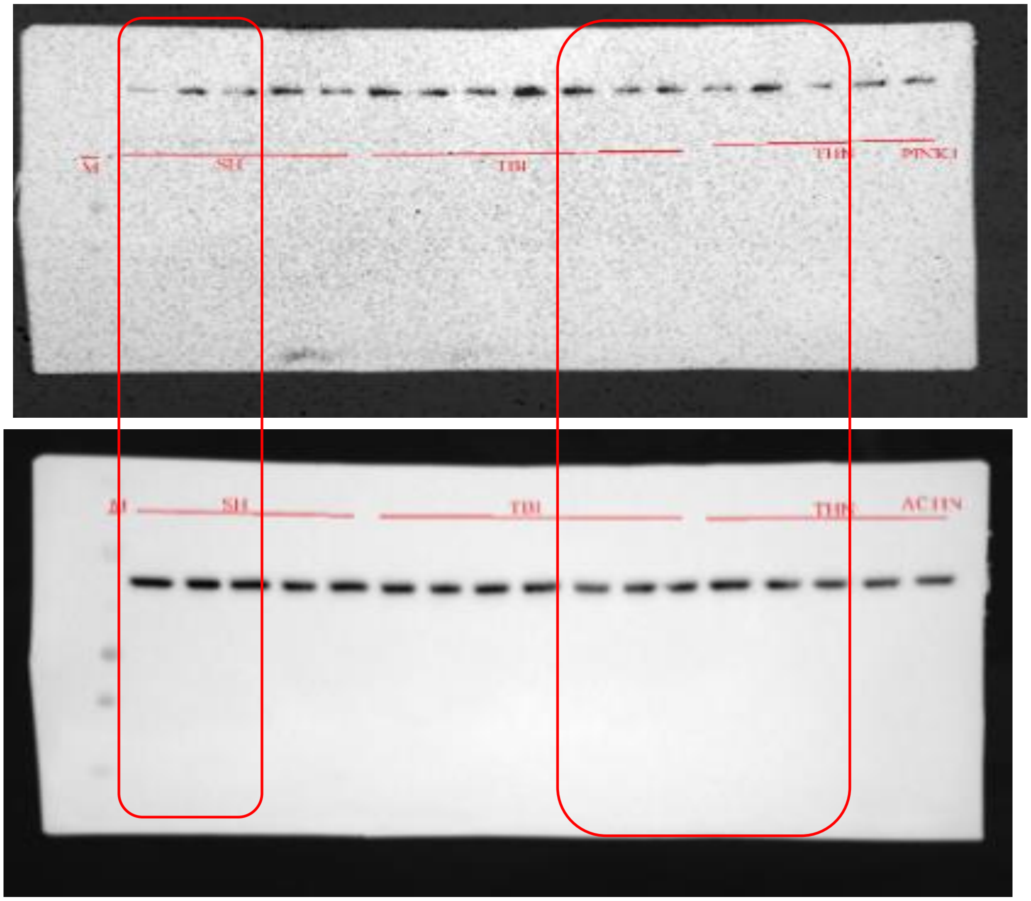

Fig.5 (E) Parkin

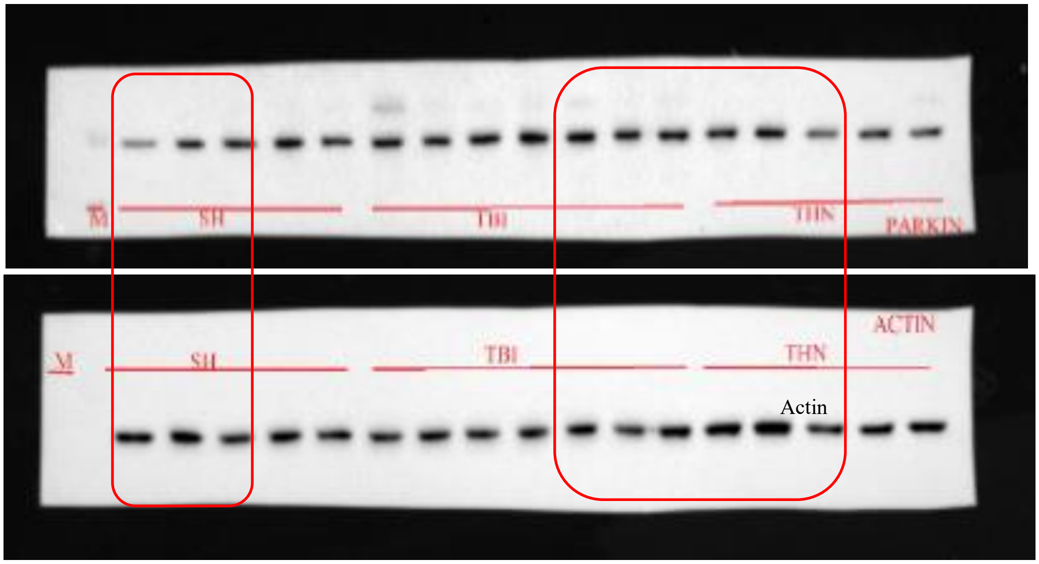

Fig.5 (F) LC3B

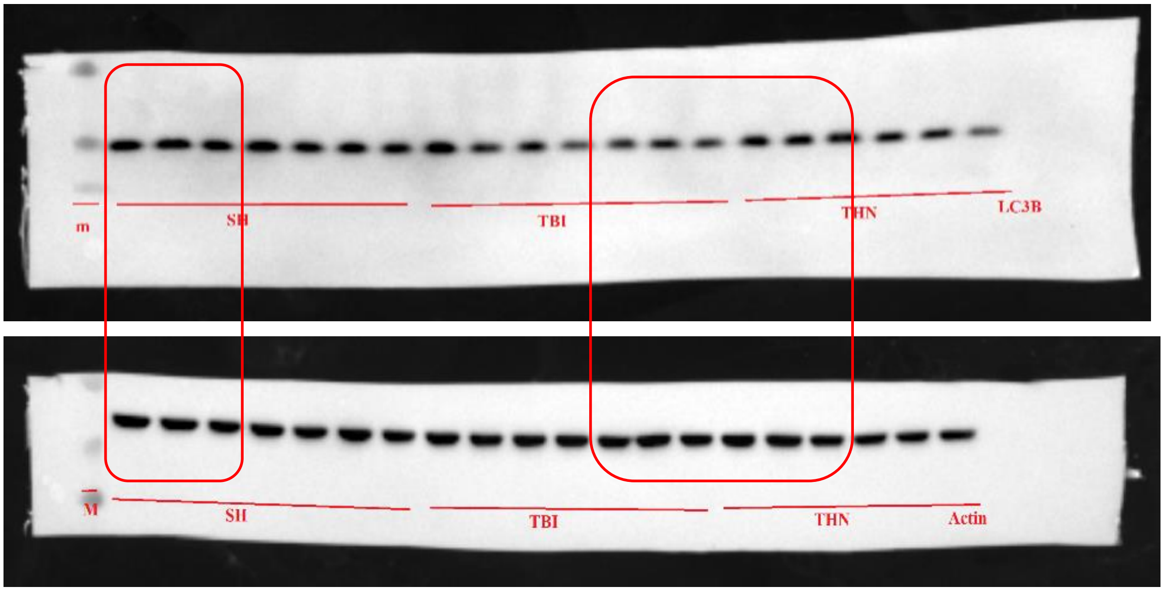

Fig.6 (A) GFAP

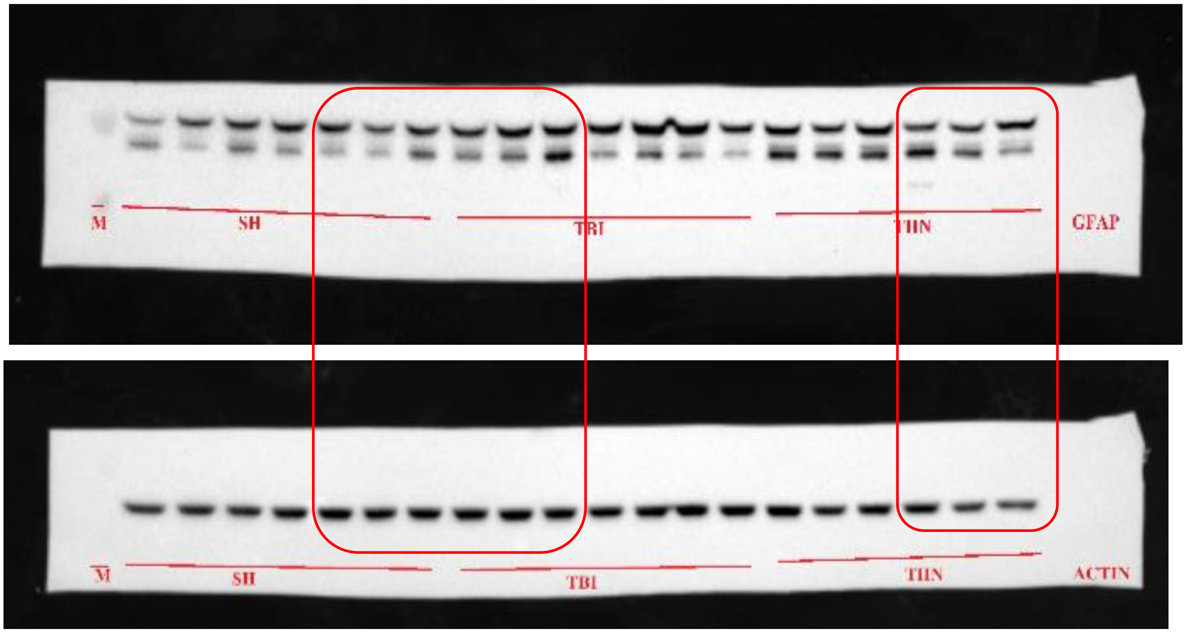

Fig.6 (B) p-STAT3

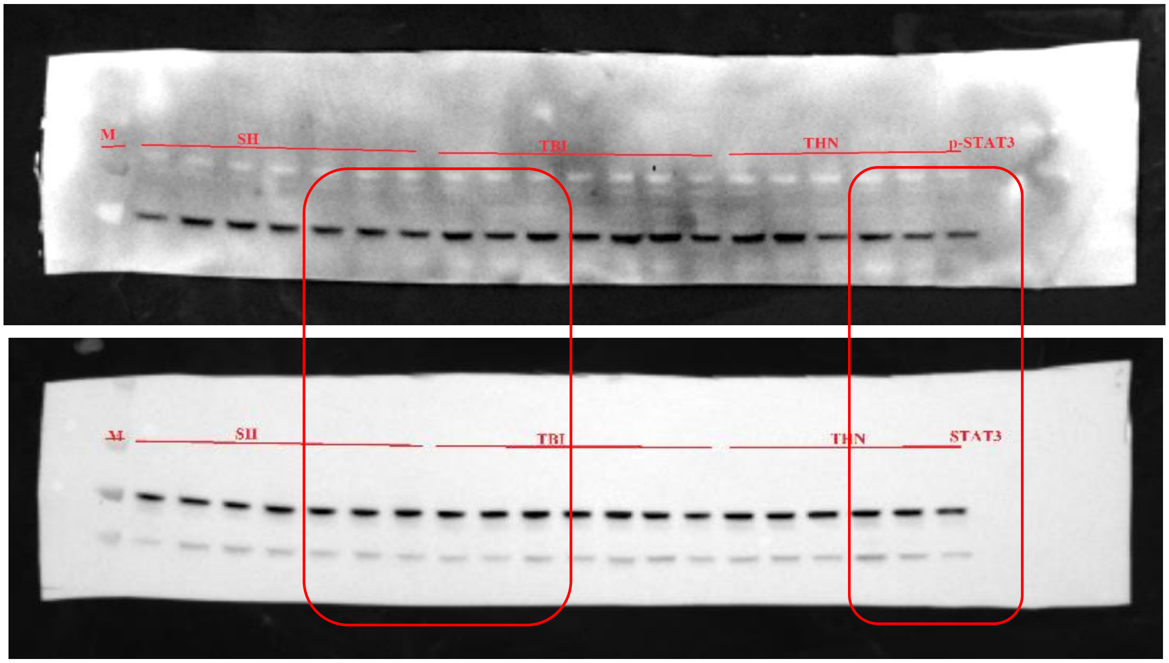

Fig.7 (A) PGC-1α

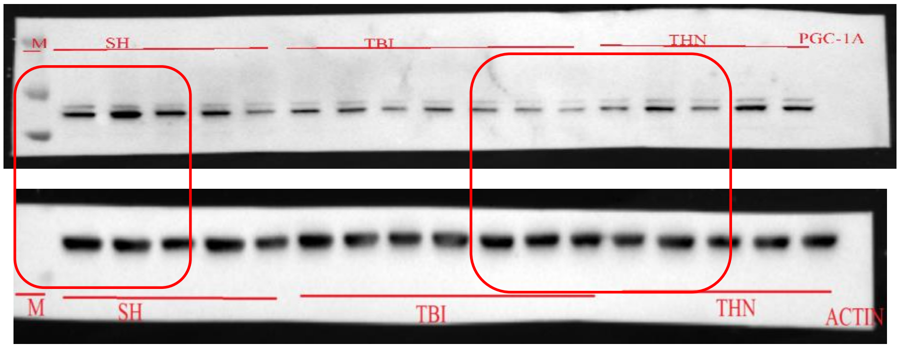

Fig.7 (B) SOD2

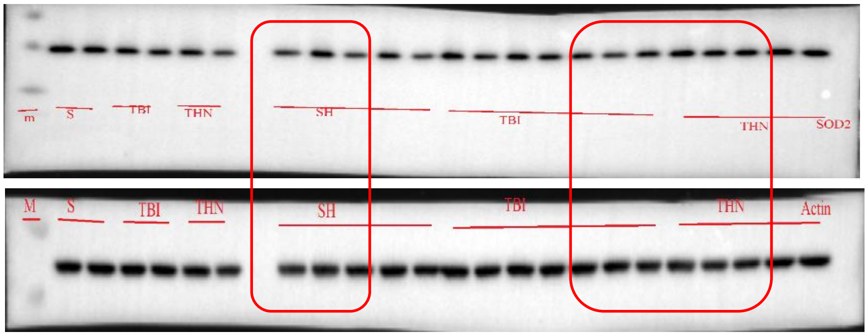

Fig.7 (C) Cleaved caspase 3 (CC3)

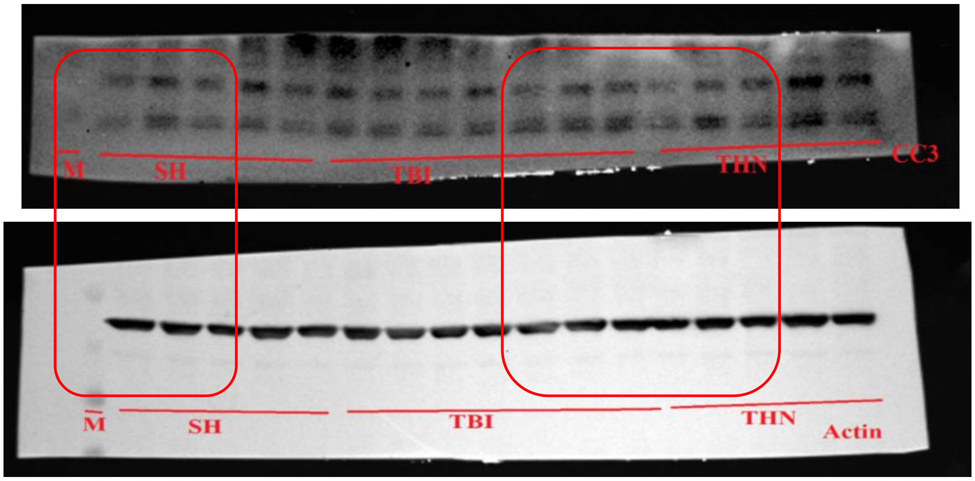

Supplement: Supplementary file 1 [file biomolecules-15-01705-s001.zip › biomolecules-3973128-supplementary.pdf]
